# Alien plant species on roadsides of the northwestern Patagonian steppe (Argentina)

**DOI:** 10.1371/journal.pone.0246657

**Published:** 2021-02-11

**Authors:** Giselle Ailin Chichizola, Sofía Laura Gonzalez, Adriana Edit Rovere

**Affiliations:** Laboratorio Ecotono, INIBIOMA, Universidad Nacional del Comahue, CONICET, Bariloche, Río Negro, Argentina; Brigham Young University, UNITED STATES

## Abstract

The introduction of alien species represents one of the greatest threats to biodiversity worldwide. Highway construction increases the dispersal and invasion of exotic plant species. This study examined the assembly process of the plant communities to determine whether the roadsides of the Patagonian steppe represent a reservoir and dispersal source of invasive exotic species. We analyzed the composition of exotic and native species and functional groups present in the established vegetation and seed banks of roadsides and reference areas nearby. The type of dispersal of exotic and native species at the roadsides was also evaluated. Total cover and that of exotic and native species was lower at the roadsides than in the reference areas; however, at the roadsides the cover and seed abundance of exotic species was higher than that of native species. In the roadsides vegetation, native shrubs such as *Acaena splendens* predominated, along with exotic perennial herbs and grasses which were mainly represented by *Rumex acetosella*. In the seed bank the predominant species were exotic perennial herbs, also represented by *R*. *acetosella*, annual exotic species such as *Epilobium brachycarpum* and *Verbascum thapsus*, and annual native species such as *Heliotropium paronychioides*. No exotic shrubs were found either at the roadsides or in the reference areas. The species at the roadsides did not present a dominant type of dispersal. The abundance of exotic species at the roadsides, both in the aboveground vegetation and the seed bank, may be due to the stressful environment and the characteristics of the species themselves, such as the ability to form seed banks. This work revealed that the roadsides of the Patagonian steppe constitute reservoirs of invasive exotic species, highlighting the importance of identifying them and controlling their spread, with a view to generating ecosystem management programs.

## Introduction

The introduction of alien species represents one of the major threats to biodiversity at a global level [1]. Exotic species can transform a habitat, affecting biodiversity on different spatial scales, contributing to species extinction [2] and creating severe challenges in terms of their management and control [3]. Their effect on biodiversity, health and the economy is so great that the control and management of invasive species has become the principal objective of numerous restoration studies [4]. Once successfully introduced outside their natural range, potentially invasive exotic plant species can propagate in the new environment if their ecological needs are met, establishing self-sustaining populations [5].

The construction of highways and roads produces considerable changes in natural communities due to plant cover elimination and soil removal, two types of disturbance that expose soils to wind and water erosion, and alter drainage, the nutrient cycle and the level of resources [6, 7]. More homogeneous habitats are thus created at the roadsides, affecting microclimatic conditions and causing considerable reduction in competition, where fast-growing ruderal species with high seed production generally become established, many of which are exotic [8, 9]. These habitats may represent a starting point for the dispersal of some exotic species, enabling them to spread and extend their distribution to non-degraded natural habitats [10]. The composition, richness and abundance of species at the roadsides therefore differ from natural areas [7, 11]. Furthermore, the lineal structure of a road and the capacity of vehicles to disperse weed seeds [12] can promote and accelerate the processes of dispersal and invasion of exotic species, extending their distribution on a regional scale [13]. Nevertheless, the colonizing potential of a species and its persistence in the invaded environment depend on its ability to overcome environmental and dispersal barriers, aspects which are related to its reproductive and dispersal characteristics [14].

The dispersal of seeds from areas with natural vegetation close to the roadsides, and their incorporation into the seed bank are important processes, since they allow natural colonization [15, 16]. In areas which have been fragmented due to the presence of roads, long-distance dispersal is a crucial process for species persistence on a regional scale and the spread of invasive exotic species [17]. A species whose fruits or seeds present structures such as plumed pappus, wings or hooks that enable them to be transported by wind (anemochory) or animals (zoochory) can extend their distribution more easily than a species that lacks propagules with these characteristics [18]. Seed dispersal also depends on seed traits (i.e., seed mass, shape and size), which enable them to be carried over long distances attached to vehicle tires and clothing [19, 20]. In European Mediterranean environments, for example, the species that colonize roadside areas mainly present fruits or seeds with anemochorous dispersal structures, other types being less common (zoochory, ballochory, barochory, etc.) [21]. Many of these species belong to the *Asteraceae* and *Poaceae* families, which produce large quantities of seeds adapted for wind dispersal [22, 23]. Since long-distance dispersal is recognized as one of the most important processes in determining invasion success [14, 24], the study of the types of dispersal of exotic species established on roadsides is essential.

Knowledge of the composition and size of the soil seed bank of disturbed areas is essential in order to understand aboveground dynamics and to predict their future floristic composition [25]. The presence of exotic species in altered areas of roadsides has consequences for seed bank composition and diversity [26, 27]. Some studies have reported that homogenized and altered habitats invaded by exotic species showed changes in composition and a decrease in the abundance of native species in the seed bank, thus impoverishing community diversity [28–30]. For example, in grasslands of Achill Island (on the west coast of Ireland), it was found that the seed banks of sectors invaded by *Gunnera tinctoria* were less diverse and abundant than in non-invaded areas [30]. Although most studies report changes in species composition, the changes in richness of native species are not always evident [31]. Characterization of the seed bank of the native, exotic, and invasive species found in disturbed environments is important for prediction of the recruiting potential of these species from the bank, as well as the future dynamics of the vegetation in the wake of new disturbances [32].

In Argentine Patagonia a great number of exotic plant species have been introduced since the beginning of the last century, principally from the Palearctic region (Holarctic Kingdom), as a result of European immigration [33]. In this region it is important to study the presence and distribution of exotic species, since the natural communities have a high level of endemism (i.e., of 2400 species, 300 are endemic, belonging to 15 endemic genera) [34]. In particular, the flora of northwest Patagonia contains approximately 300 species of exotic plants, equivalent to 20% of the total number of species in the region [35]. A study of floristic composition in the north of Argentine Patagonia, carried out on a west-east precipitation gradient that included areas of forest, shrubland and steppe, revealed that the shrubland and steppe had the highest presence of exotic species, the most frequent being *Bromus tectorum*, *Cerastium arvense* and *Rumex acetosella* [36]. A large number of studies in the world have assessed the influence of roads on exotic plant dispersal and establishment in aboveground vegetation [9, 11, 13, 37, 38], but soil seed bank on roadsides have been less examined [27, 39]. In particular these topics have been under-researched in Patagonian steppe roadsides [26, 40].

Assessing the functional composition of communities enables us to compare assembly processes between studies in different regions and on different scales [41, 42]. Classification of plant species into functional groups according to their life cycle and life form can be applied to the study of ecological processes, such as the dispersal, colonization and establishment of species at roadsides [8]. In general, the species that colonize roadsides are short-lived, monocarpic annual or perennial species [23, 37]. In northwest Patagonia, the response of different functional groups to disturbances such as fire, volcanic eruption, and drought have been much studied [43, 44]; however, in this region no research has been carried out on colonization of the roadside habitat by the different functional groups. Analyzing functional groups enables to draw more general conclusions on assembly process compared to species-specific analysis.

The objective of the present study was to examine the assembly process of the plant communities to determine whether the roadsides of the steppe in northwest Patagonia constitute a reservoir and dispersal source of invasive exotic species. To this end, the composition of the native and exotic species and functional groups in the established vegetation and seed banks were analyzed for the roadsides and for neighboring areas. The type of dispersal of the exotic and native species present at the roadsides was also studied. Our hypothesis is that the altered areas at the roadsides present conditions for the colonization and later spread of exotic species. We predict that at the roadsides the vegetation cover and seed density in the soil seed bank of exotic species will be higher than the cover and seed density of native plants. Therefore, low similarity in species composition of vegetation and seed bank between roadsides and neighboring areas is expected. With respect to functional groups, we predict that the cover and seed density of annual and short-lived perennial herbs will be higher at the roadsides than in neighboring areas. We predict that species with anemochorous dispersal will be more successful at roadsides, because their seeds can be dispersed over long distances. The knowledge of the floristic composition of roadside vegetation allows the identification of exotic species to be controlled, as well as pioneer native species suitable for reintroduction that provides information for the design of specific restoration protocols [16, 45,].

## Materials and methods

### Study area

**Fig 1:**
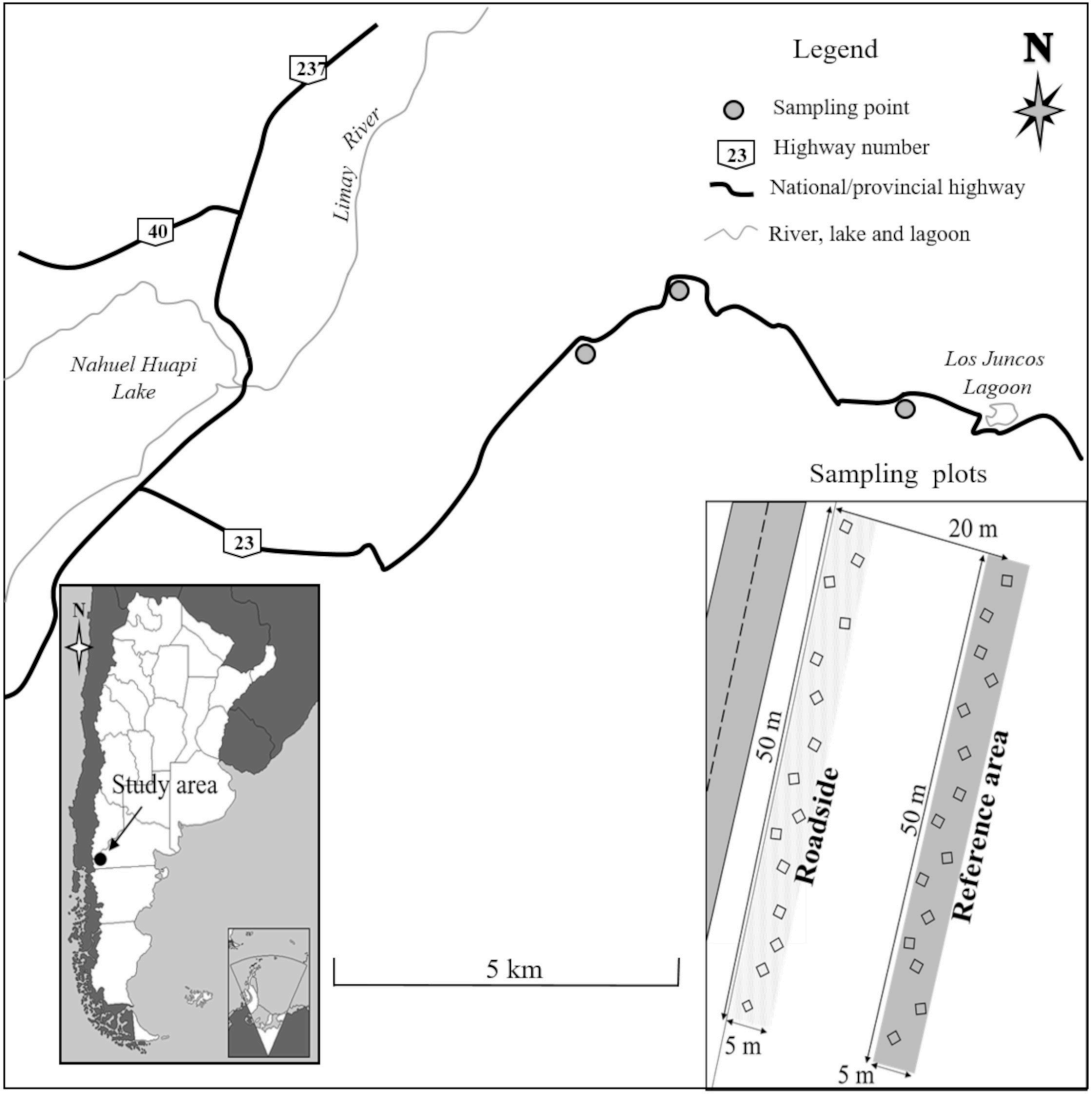
Location of study area and sketch of sampling design.

Fieldwork was carried out along a 6 km fraction of National Highway 23 and contiguous areas of herbaceous-shrub steppe grassland in northwest Patagonia, which is situated 30 km east of the city of San Carlos de Bariloche, within San Ramón Ranch (Argentina) (Fig 1). This project was conducted using the field permit given by the San Ramón Ranch managers John Belcher and Leandro Ballerini.

Climate in the region is semiarid and temperate, with average annual precipitation of 574.4 mm (precipitation is seasonally distributed, with 60% falling in autumn-winter) and an average annual temperature of 8.6°C (non-published data from the meteorological station on San Ramon ranch). Strong W-NW winds are frequent all year round, with a mean speed of 30 km/h [46]. The dominant soil types are moderately developed (Haploxerolls) with a loamy-sandy texture and superficial horizons containing moderate organic matter [47]. The typical vegetation consists mainly of a matrix dominated by the grasses *Pappostipa speciosa* and *Festuca pallescens*, and by scattered shrubs such as *Azorella prolifera* and *Senecio bracteolatus*. Shrubs and grasses are intermixed with small native and exotic herbs [43].

### Sampling design

Three sampling points were selected along the highway, separated from one another by about 2 km. At each point we selected two different plots 5 m wide and 50 m long parallel to the road. The first one (Roadside plot) was adjacent to the road and had similar environmental conditions (north orientation, slope less than 45°), and the second one (Reference area plot) was located 20 m from the road within the grassland (Fig 1). Typically, these highways have roadsides with an artificial slope caused by ground levelling during road construction, and fencing that separates it from the surrounding area. Sampling was performed on roadsides that had not been previously restored or rehabilitated, and in the neighboring steppe matrix where livestock farming is practiced. Extensive livestock farming has been the most important productive activity in the region since the beginning of last century [48].

### Vegetation sampling

In December of 2016 (spring), at each sampling point 15 quadrats of 1m^2^ were placed at random in each plot (roadside and reference area) to determine species composition, cover, and richness (3 sampling points x 2 plots x 15 quadrats) (Fig 1). Species cover was estimated in each quadrat using the Braun-Blanquet scale [49]. In addition, all species present within a distance of 1 m from each side of the quadrat were recorded.

### Seed bank assessment

The seed bank was assessed at both plots in April 2017 (in autumn, after seed dispersal but before germination). At each sampling point, 10 soil samples were taken at random per plot (roadside and reference area), giving a total of 60 soil samples. Each sample measured 8.5 cm in diameter and 3 cm in depth. The samples were stratified in a refrigerator (at 5°C) for 3 months. After this time, they were sieved to eliminate stones and plants remaining before being placed in containers on a substrate of sterile sand (for 2 days at 100°C) to promote water drainage [43, 50]. Finally, they were put in a greenhouse under conditions of controlled irrigation. Over the next 10 months the seedlings were counted and identified every week using a regional seedling identification guide [51]. Seedlings which could not be identified were transplanted for later identification through observation of their reproductive structures [52].

### Data analysis

Plants from the vegetation and seed banks were identified at species level and classified according to their origin (native, endemic and exotic) in the Southern Cone, composed of the countries Argentina, Chile, Uruguay, Paraguay and southern Brazil [35, 53]. The exotic species were classified as invasive using The Global Invasive Species Database [54], Global Register of Introduced and Invasive Species [55], and local bibliography [56]. The frequency of different botanical families (exotic plus native) present in the vegetation and seed bank was analyzed for the roadsides (RS) and reference areas (RA), as well as exotic and native species separately for the RS and RA. The species were classified into three functional groups based on their life form and life cycle: annual/biannual herbs and grasses (Group I); perennial herbs and grasses (Group II); and shrubs (Group III) [43]. Finally, the type of fruit or seed dispersal (anemochory, autochory and zoochory) was determined for each species [36, 52].

To compare total cover and the cover of native and exotic species, functional groups, and the most abundant species (distinguishing between native and exotic) between RS and RA and for each plot, we used generalized linear mixed models (glmmTMB—R package “glmmTMB”) with a Beta distribution [57, 58]. In these models cover was included as a response variable. In the comparisons between RS and RA, plot (roadside, reference area) was taken as the explanatory variable, and for comparisons within each plot, origin (native, exotic), functional groups, or the most abundant species were taken as explanatory variables, and the sampling points as a random effect.

To compare mean species richness of the native and exotic species in the vegetation and the seed bank, total seed density and the seed density of exotic and native species, functional groups and the most abundant species (distinguishing between native or exotic) between RS and RA and for each plot, we used generalized linear mixed models (bglmer—R package “blmer”) with a Poisson distribution [57, 58]. The number of species and seeds were taken as response variables in the models. For the comparisons between RS and RA the plot was taken as the explanatory variable and the sampling points as a random effect. Origin, functional groups or the most abundant species were considered as explanatory variables for comparisons within each plot, and the sampling points as the random effect.

For vegetation and seed bank response variables, the Bonferroni tests were used for posteriori multiple comparisons between functional groups and the most abundant species. For these analyses the “glht” function of the “multcomp” R package was used [59].

All the analyses were performed using the open source software R, version 4.0.2 [60].

The Sørensen index was used to calculate similarity of species composition between the vegetation and the soil seed bank in RS and RA. The following formula was used: *2a/(2a + b + c)*, where *a* is the number of species common to both the vegetation and the seed bank, *b* is the number of species exclusive to the vegetation, and *c* is the number of species exclusive to the seed bank. Furthermore, species similarity was calculated between the roadsides and the reference areas for the vegetation and the seed bank. In this case *a* is the number of species common to both the roadsides and the reference areas, *b* is the number of species exclusive to the roadsides and *c* is the number of species exclusive to the reference areas [49].

The percentage of exotic and native anemochorous and non-anemochorous (autochory plus zoochory) species present in the vegetation and in the seed bank at the roadsides were analyzed using a Chi Square test.

The species present in the plots, but recorded outside the sampling quadrats, were considered only for analysis of total richness, similarity of composition of the vegetation and type of dispersal.

## Results

### Floristic composition

A total of 53 plant species belonging to 47 genera and 25 families were found in the vegetation and seed bank at the roadsides (RS) and the reference areas (RA). Of this total 33 were native species (62%), of which 23 are endemic (43%), and 20 are exotic species (38%). Of the exotic species, 12 are invasive (60%) (Table 1). The most represented families were *Asteraceae*, *Poaceae* and *Rosaceae*, and the families with the highest number of exotic species were *Asteraceae*, with 6 species, and *Poaceae* with 4 (Table 1).

**Table 1:** Species present at the roadsides and nearby reference areas in Patagonian steppe.

Scientific name (botanic family)	Origin	Dispersion	RS		RA	
			VEG	SB	VEG	SB
**Group I: Annual/biannual herbs and grasses**						
*Apera interrupta* (Poaceae)	Exo	Zoo	X		X	
*Boopis gracilis* (Calyceraceae)	Nat*	Aut	X		X	
*Bromus tectorum* (Poaceae)	Exo*	Ane	X		X	
*Carduus thoermeri* (Asteraceae)	Exo*	Ane	X	X	X	
*Chenopodium scabricaule* (Chenopodiaceae)	Nat	Aut	p			
*Collomia biflora* (Polemoniaceae)	Nat	Aut	X			
*Conyza lechleri* (Asteraceae)	Nat*	Ane			X	
*Draba verna* (Brassicaceae)	Exo	Ane	X	X	X	X
*Epilobium brachycarpum* (Onagraceae)	Exo	Ane	X	X	X	X
*Erodium cicutarium* (Geraniaceae)	Exo	Zoo	X			
*Festuca australis* (Poaceae)	Nat*	Ane	X	X	X	
*Heliotropium paronychioides* (Boraginaceae)	Nat*	Aut	X	X		X
*Holosteum umbellatum* (Caryophyllaceae)	Exo	Aut	X	X		
*Lactuca serriola* (Asteraceae)	Exo*	Ane		X		
*Montiopsis polycarpioides* (Montiaceae)	Nat*	Aut				X
*Nicotiana linearis* (Solanaceae)	Nat	Aut	p	X		
*Sisymbrium altissimum* (Brassicaceae)	Exo	Ane	X	X	X	X
*Tragopogon dubius* (Asteraceae)	Exo	Ane			X	
*Tripleurospermum inodorum* (Asteraceae)	Exo	Ane	X	X		
*Triptilion achilleae* (Asteraceae)	Nat*	Ane	X		X	
*Verbascum thapsus* (Scrophulariaceae)	Exo*	Aut	X	X		X
**Group II: Perennial herbs and grasses**						
*Acaena magellanica* (Rosaceae)	Nat	Zoo	p			
*Acaena pinnatifida* (Rosaceae)	Nat*	Zoo	X	X	X	
*Astragalus palenae* (Fabaceae)	Nat*	Ane	X			
*Bromus setifolius* (Poaceae)	Nat*	Ane	X			
*Carex andina* (Cyperaceae)	Nat*	Aut		X		
*Euphorbia collina* (Euphorbiaceae)	Nat*	Aut	X		X	
*Festuca pallescens* (Poaceae)	Nat*	Ane	X			
*Holcus lanatus* (Poaceae)	Exo*	Ane	p			
*Hordeum comosum* (Poaceae)	Nat	Ane	X		X	
*Hypochaeris radicata* (Asteraceae)	Exo*	Ane		X		X
*Juncus stipulatus* (Juncaceae)	Nat	Aut		X		
*Pappostipa humilis* (Poaceae)	Nat*	Ane			X	
*Pappostipa speciosa* (Poaceae)	Nat	Ane	X		X	
*Phacelia secunda* (Boraginaceae)	Nat	Aut	X		X	
*Plantago lanceolata* (Plantaginaceae)	Exo*	Aut	X	X		
*Poa ligularis* (Poaceae)	Nat*	Ane	X		X	
*Poa pratensis* (Poaceae)	Exo*	Ane		X		
*Rhodophiala mendocina* (Amaryllidaceae)	Nat	Ane	X		X	
*Rumex acetosella* (Polygonaceae)	Exo*	Aut	X	X	X	X
*Sisyrinchium arenarium* (Iridaceae)	Nat*	Aut	X		X	
*Taraxacum officinale* (Asteraceae)	Exo*	Ane		X	X	
*Verónica serpyllifolia* (Plantaginaceae)	Exo*	Aut		X		
**Group III: Perennial shrubs**						
*Acaena splendens* (Rosaceae)	Nat	Zoo	X	X	X	X
*Azorella prolifera* (Apiaceae)	Nat	Ane	X		X	
*Baccharis linearis* (Asteraceae)	Nat*	Ane				X
*Baccharis neaei* (Asteraceae)	Nat*	Ane		X		
*Berberis microphylla* (Berberidaceae)	Nat*	Zoo			p	
*Ephedra chilensis* (Ephedraceae)	Nat*	Aut			p	
*Fabiana imbricata* (Solanaceae)	Nat*	Aut				X
*Grindelia anethifolia* (Asteraceae)	Nat*	Ane	X		X	
*Rosa rubiginosa* (Rosaceae)	Exo*	Zoo	p			
*Senecio bracteolatus* (Asteraceae)	Nat*	Ane	X	X	X	X

Scientific name (botanic family), origin of species (Exo = exotic, Nat = native, Nat* = endemic), species classified as invasive exotic (Exo*) and type of dispersion (Ane = Anemochory, Aut = Autochory, and Zoo = Zoochory). (X) indicates that the species was found in the vegetation (VEG) and/or soil seed bank (SB) of the roadsides (RS) and/or reference areas (RA). (p) indicates that the species was present in the plot but not inside the sampling quadrats. Species were grouped into functional groups (I = annual/biannual herbs and grasses, II = perennial herbs and grasses, III = perennial shrubs).

**Fig 2:**
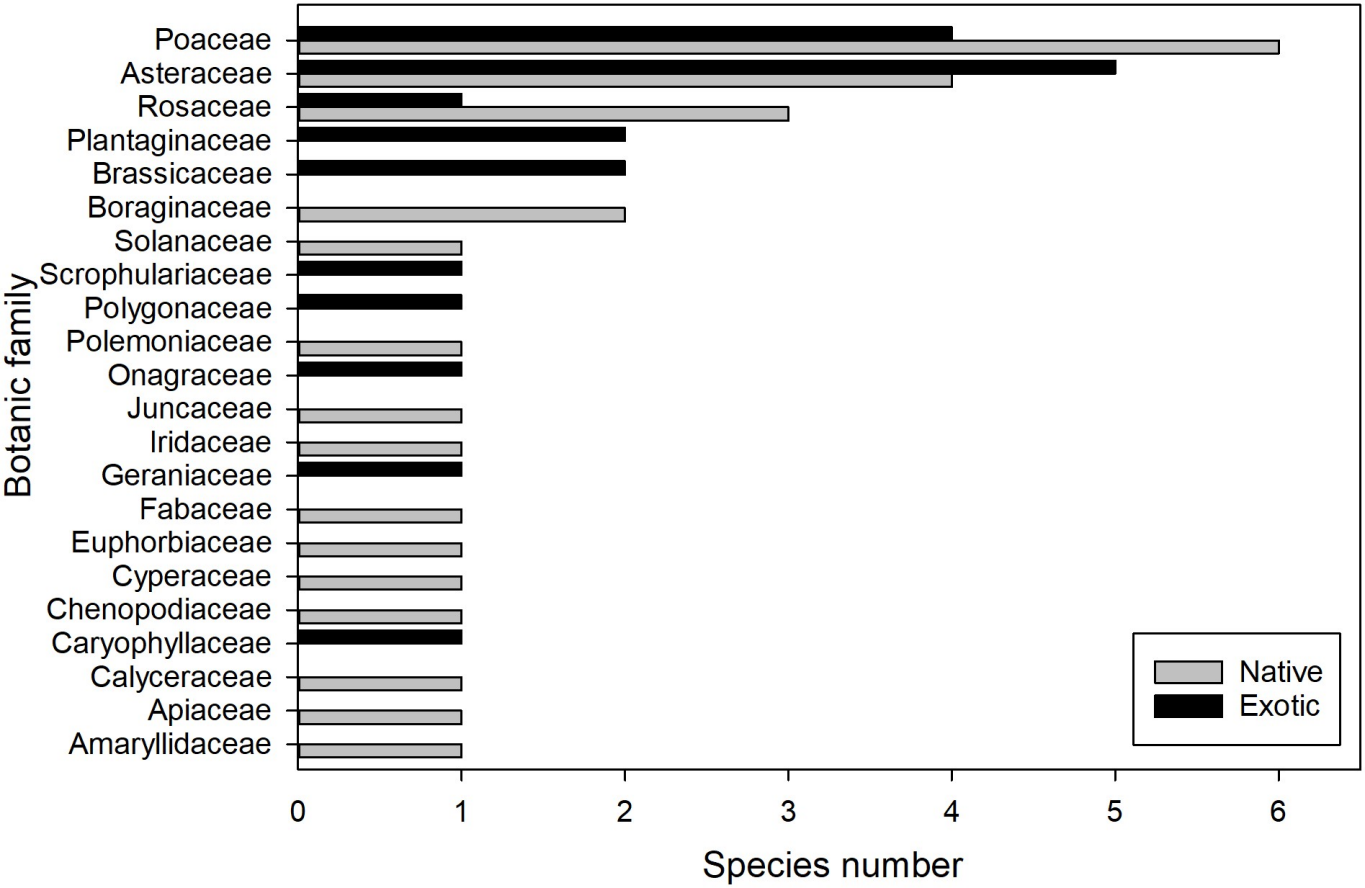
Species by botanic family at roadsides. Number of exotic and native species by botanic family in the vegetation and the soil seed bank at the roadsides.

In the vegetation and seed bank of RS a total of 45 species were found, belonging to 22 families. Of this number 19 were exotic species (42%), and 12 of these are invasive (63%) (Table 1; Fig 2). The *Asteraceae*, *Poaceae*, *Brassicaceae* and *Plantaginaceae* families accounted for 68% of the exotic species, most of which are invasive (e.g., *Carduus thoermeri*, *Taraxacum officinale* and *Bromus tectorum*) (Fig 2; Table 1). In RA a total of 35 species were found, belonging to 17 families, where 11 species (31%) were exotic, and of this number 6 are invasive (55%). The *Asteraceae*, *Poaceae* and *Brassicaceae* families were the most common (Table 1).

### Richness, total cover and cover of functional groups

**Fig 3:**
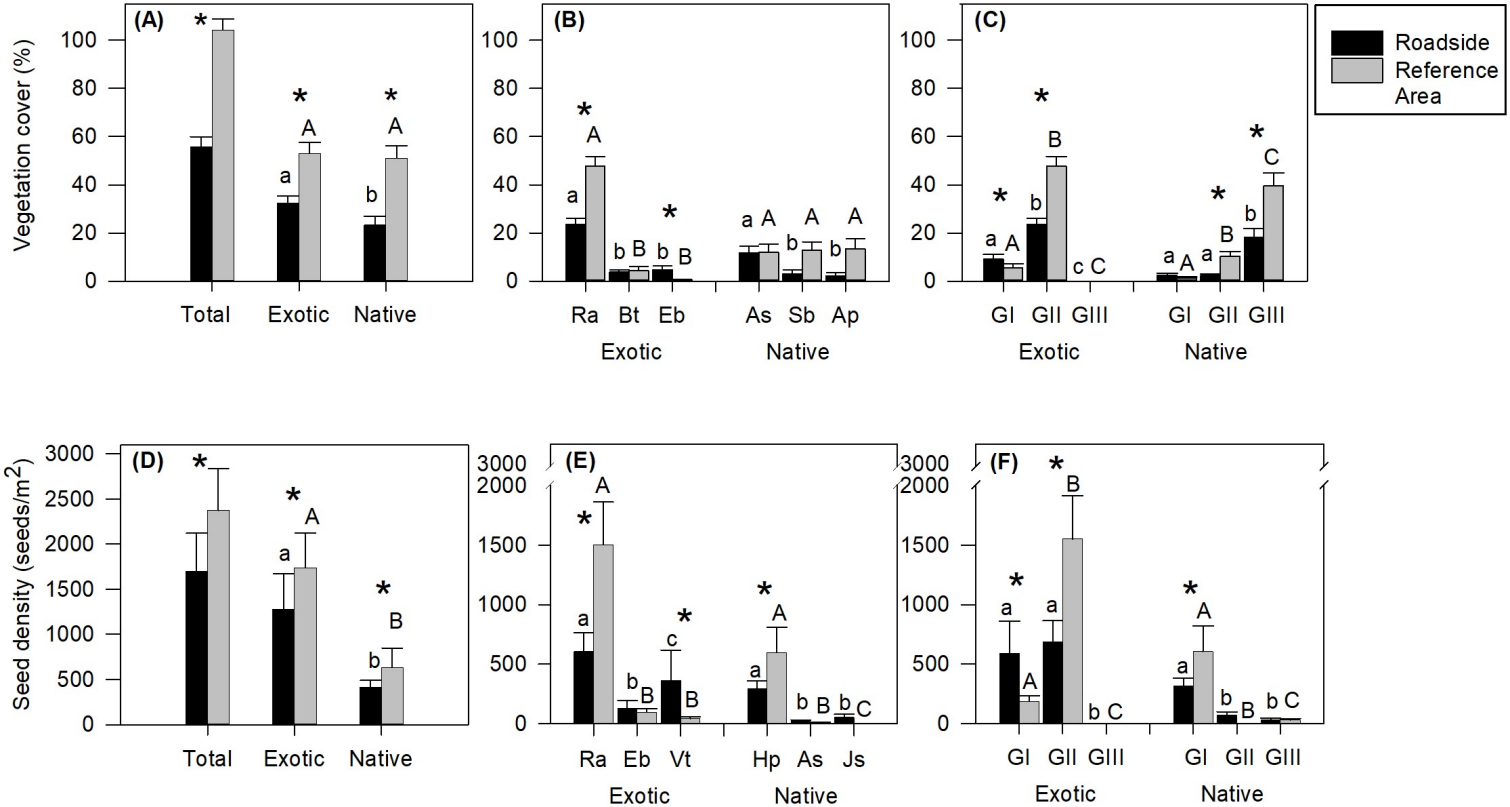
Vegetation cover, density of seeds in the seed bank and functional groups in the roadsides and references areas. Mean vegetation cover ± SE at roadsides and reference areas of: (A) total, exotic and native species; (B) the most abundant exotic species: *Rumex acetosella* (Ra), *Bromus tectorum* (Bt), *Epilobium brachycarpum* (Eb), and native species: *Acaena splendens* (As), *Senecio bracteolatus* (Sb) and *Azorella prolifera* (Ap); (C) functional groups (GI = annual/biannual herbs and grasses, GII = perennial herbs and grasses, GIII = perennial shrubs). Mean seed density ± SE at roadsides and reference areas of: (D) total, exotic and native species; (E) the most abundant exotic species: *R*. *acetosella* (Ra), *E*. *brachycarpum* (Eb), *Verbascum thapsus* (Vt), and native species: *Heliotropium paronychioides* (Hp), *A*. *splendens* (As) and *Juncus stipulatus* (Js); (F) functional groups. *indicates significant differences between cover (total, exotic and native), seed density (total, exotic and native), vegetation cover and seed density of the most abundant species and functional groups between roadsides and reference areas (*P* < 0.05). Lower-case letters indicate significant differences between vegetation cover and seed density of native and exotic species, vegetation cover and seed density of the most abundant species and functional groups at roadsides (*P* < 0.05). Capital letters represent significant differences between vegetation cover and seed density of native and exotic species, vegetation cover and seed density of the most abundant species and functional groups at reference areas (*P* < 0.05).

The mean richness of the vegetation was similar in RS (6.1 ± 0.7) and RA (6.6 ± 0.8) (*X*^*2*^ = 0.9, Df = 1, *P* = 0.34). The mean richness of exotic species was also similar in RS (2.5 ± 0.5) and RA (2.1 ± 0.4) (*X*^*2*^ = 1.7, Df = 1, *P* = 0.19), while the mean richness of native species (3.6 ± 0.4) was lower in RS than in RA (4.5 ± 0.5) (*X*^*2*^ = 4.8, Df = 1, *P* = 0.03). RS had lower total cover (*X*^*2*^ = 42.5, Df = 1, *P* < 0.001), and lower cover of exotic (*X*^*2*^ = 9.6, Df = 1, *P* = 0.002) and native species (*X*^*2*^ = 19.1, Df = 1, *P* < 0.001) than RA. RS had higher cover of exotic than native species (*X*^*2*^ = 4.6, Df = 1, *P* = 0.03), whereas in the RA the cover of exotic and native species was similar (*X*^*2*^ = 0.2, Df = 1, *P* = 0.68) (Fig 3A). As regards the most abundant exotic species, the perennial herb *Rumex acetosella* had higher cover at RA than RS (*X*^*2*^ = 15, Df = 1, *P <* 0.001), whereas the annual herb *Epilobium brachycarpum* had higher cover at RS than RA(*X*^*2*^ = 7.9, Df = 1, *P =* 0.005) and the annual grass *B*. *tectorum* had a similar cover value in RS and RA (*X*^*2*^ = 0.1, Df = 1, *P =* 0.79) (Fig 3B). *Rumex acetosella* had the highest cover of all the exotic species in RS (*X*^*2*^ = 72, Df = 2, *P <* 0.001) and RA (*X*^*2*^ = 131.6; Df = 2, *P <* 0.001) (Fig 3B). Of the most abundant native species, *Acaena splendens* had the highest cover in comparison with the other species at RS (*X*^*2*^ = 10.84, Df = 2, *P =* 0.004) (Fig 3B). In RA the cover of *A*. *splendens*, *Azorella prolifera* and *Senecio bracteolatus* (the most abundant native species) was not significantly different (*X*^*2*^ = 0.32, Df = 2, *P* = 0.85) (Fig 3B). The cover of *A*. *splendens* (*X*^*2*^ = 0.04, Df = 1, *P* = 0.84), *A*. *prolifera* (*X*^*2*^ = 1.74, Df = 1, *P* = 0.18) and *S*. *bracteolatus* (*X*^*2*^ = 2.85, Df = 1, *P* = 0.09) was not significantly different between RS and RA (Fig 3B).

Cover of the exotic species belonging to group I (annual/biannual herbs and grasses) (*X*^*2*^ = 4, Df = 1, *P =* 0.04) was greater in RS than in RA. With regard to exotic (*X*^*2*^ = 15, Df = 1, *P <* 0.001) and native (*X*^*2*^ = 11.7, Df = 1, *P <* 0.001) species belonging to groups II (perennial herbs and grasses) and native species belonging to group III (shrubs) (*X*^*2*^ = 7.1, Df = 1, *P =* 0.008), cover was greater in RA than in RS (Fig 3C). Exotic species belonging to group II contributed to most cover in RS (*X*^*2*^ = 19.9, Df = 1, *P <* 0.001) and RA (*X*^*2*^ = 62.8, Df = 1, *P <* 0.001) (Fig 3C). In RS native species from group III were the second group that contributed to most cover (*X*^*2*^ = 22.1, Df = 2, *P <* 0.001). In RA native species from group III had more cover than the other native species groups, group II species providing more cover than group I species (*X*^*2*^ = 46.9, Df = 2, *P <*0.001) (Fig 3C). No exotic shrubs were found in the quadrats of either plot, although the presence of *Rosa rubiginosa* was observed at RS.

### Richness, total seed density and seed density of functional groups

Mean richness of the seed bank was similar in RS (3 ± 0.6) and RA (2.3 ± 0.4) (*X*^*2*^ = 3.3, Df = 1, *P* = 0.07). At RS the mean richness of exotic (2 ± 0.4) and native (1 ± 0.3) species was similar to the mean of exotic (1.6 ± 0.3) and native (0.6 ± 0.2) species at RA (*X*^*2*^ = 1.3 Df = 1, *P* > 0.05; *X*^*2*^ = 2.5 Df = 1, *P* > 0.05, respectively).

The total density of seeds (*X*^*2*^ = 18.9, Df = 1, *P <* 0.001), and the density of seeds from exotic (*X*^*2*^ = 11.7, Df = 1, *P <* 0.001) and native (*X*^*2*^ = 7.6, Df = 1, *P =* 0.006) species in RS was lower than in RA (Fig 3D). In RS and RA the number of seeds of exotic species was higher than the number of seeds of native species (RS: *X*^*2*^ = 67.7, Df = 1, *P <* 0.001; RA: *X*^*2*^ = 80.7, Df = 1, *P <* 0.001) (Fig 3D). With respect to the most abundant exotic species, a higher number of *R*. *acetosella* seeds was registered in RA than in RS (*X*^*2*^ = 61.1, Df = 1, *P* < 0.001), and a higher number of seeds of the biannual herb *Verbascum thapsus* was found in RS than in RA (*X*^*2*^ = 30.5, Df = 1, *P* < 0.001), while a similar number of seeds of the annual herb *Epilobium brachycarpum* was found in both plots (*X*^*2*^ = 1, Df = 1, *P* = 0.32) (Fig 3E). *Rumex acetosella* was the species that contributed most seeds to the bank in RS (*X*^*2*^ = 45.8, Df = 2, *P* < 0.001) and RA (*X*^*2*^ = 197.3, Df = 2, *P* < 0.001). In RS the number of *V*. *thapsus* seeds was higher than that of *E*. *brachycarpum* (*P* < 0.001) (Fig 3E). Of the most abundant native species, *Heliotropium paronychioides* contributed the largest number of seeds to the bank in RS (*X*^*2*^ = 41.5, Df = 2, *P* < 0.001) and RA (*X*^*2*^ = 21.5, Df = 1, *P* < 0.001) (Fig 3E), whereas the seed density of this species was higher in RA than in RS (*X*^*2*^ = 17.2, Df = 1, *P* < 0.001). A similar number of seeds of the shrub *Acaena splendens* was found in RS and RA (*X*^*2*^ = 0.9, Df = 1, *P =* 0.34), while seeds of the perennial herb *Juncus stipulatus* were recorded only in RS. Both these species contributed a low density of seeds to the banks.

The number of seeds from exotic species of group I (*X*^*2*^ = 31.25, Df = 1, *P* < 0.001) was higher in RS than RA. Seeds of native species from group II were found only in RS. The numbers of seeds of exotic species from group II (*X*^*2*^ = 50.13, Df = 1, *P* < 0.001) and native species from group I (*X*^*2*^ = 14.91, Df = 1, *P* < 0.001) were higher in RA than in RS (Fig 3F). At RS the seed bank contained mainly exotic species from groups I and II (*X*^*2*^ = 1.2, Df = 1, *P* = 0.28), and native species from group I (*X*^*2*^ = 43.2, Df = 2, *P* < 0.001). At RA exotic species from group II were more abundant in the seed bank (*X*^*2*^ = 127.7, Df = 1, *P* < 0.001), along with native species from group I (*X*^*2*^ = 44.2, Df = 1, *P* < 0.001) (Fig 3F). In the seed bank no seeds were found of exotic species from group III in RS and RA, and the only native species found from group III were *A*. *splendens*, *Baccharis linearis*, *S*. *bracteolatus* and *Fabiana imbricata* at RA (Table 1).

The species similarity between vegetation and seed bank at RS according to the Sørensen index was 47%, while at RA the similarity value was 29%. Similarity of species in the vegetation of RS and RA was 71%, while for the seed bank this value was 51%.

With regard to the types of seed dispersal, the native and exotic species in the vegetation and the seed bank at the roadsides presented no difference in type of dispersal (*X*^*2*^ = 0.19, Df = 1, *P* = 0.6). Exotic and native anemochorous species accounted for 24% and 29% of the species richness, respectively. Exotic and native non-anemochorous species (autochorous and zoochorous) represented 18% and 29% of the species richness, respectively (Table 1).

## Discussion

This study contributes to the research in invasion ecology and the role of roads on the dispersal of exotic species by providing spatial (roadside vs reference area) and temporal (vegetation vs seed bank) information for assessing plant invasions in the northwest Patagonian region. Our study found that the predominant plants in the vegetation and seed bank were exotic perennial and annual herbs with different types of dispersal. It also found that there was limited expansion of exotic species further from the roads, probably attributed to the low frequency of disturbance that generate resource release (e.g. space and light) and to species traits (e.g. competitive ability).

In this study, the majority of exotic species at the roadsides belonged to the *Asteraceae* and *Poaceae* families, results that coincide with other studies carried out on the roadsides in southern Chile [22] and in the center of Argentina [61]. These families contribute a large number of exotic invasive species to the world’s flora [62]. Many of these invader species have spreading strategies related to efficient long-distance wind or animals dispersal [14, 40, 63].

In this work it was found that the total vegetation cover was lower at the roadsides than in the reference areas, in agreement with another study in the Patagonian region [26]. Low cover at roadsides is frequently seen, due to the elimination of surface soil layers and the reduction in organic material brought about by road construction [64]. The new substrates at the roadsides are susceptible to erosion, with changes in water availability that hamper colonization [37, 65]. As expected, in this study the vegetation cover of exotic species was higher than native species at the roadsides. This may be a result of the environmental conditions at the roadsides (e.g., greater availability of light, bare soil, soil texture) [66, 67], which generate habitats suitable for the establishment, growth and reproduction of exotic plants to the detriment of native species [37, 23]. Other studies reported that the disturbance of the roadside and the movement of vehicles favor the entry of propagules of exotic species [68, 69]. These factors may also affect species richness, since that roadside vegetation presented lower native species richness than the reference area, coinciding with other studies [26, 61].

The high cover of exotic species in the reference areas was due to the marked dominance of the perennial herb *Rumex acetosella*, which was responsible for the high cover of the exotic perennial herbs and grasses functional group in the roadsides and reference areas. *Rumex acetosella* first came into northern Patagonia with domestic cattle over 100 years ago, and its dominance and permanence could be due to its double strategy of bud and seed bank regeneration and its high competitivity [70, 71]. This ruderal species dominates the steppe gaps, occupying safe microsites for the regeneration of many species, including the dominant matrix and competes for the resources, having a negative effect on the growth of other species [71, 72]. It is likely that the dominance of *R*. *acetosella* in the gaps may limits the colonization and establishment of other exotic species, producing invasional interference [73].

Despite the dominance of *R*. *acetosella* at the roadsides, the annual grass *Bromus tectorum* and annual herb *Epilobium brachycarpum*, both exotic, contributed greatly to the vegetation cover of the roadsides, perhaps due to the greater availability of space because of the low plant cover. Both annual herb and grass appear to depend on intense disturbance (i.e., removal of soil, fire, grazing) to spread into less degraded sites. It is documented that *B*. *tectorum* spread from the roadsides to the interior of the natural ecosystem [40] and *E*. *brachycarpum* is frequently found in degraded environments [52], while in little-disturbed natural habitats it presents low cover [43]. The high germination potential, abundant seed production and high dispersal capacity by wind or animals of both species could favor their establishment in disturbed zones [74–76]. Both species have been registered as invasive in other regions of the world [74, 77], having an important impact on native communities. For example, *B*. *tectorum* alters the nutrient cycle, water availability and fire frequency, and may exclude native species [74]. Therefore, their population dynamics and effects on native ecosystems need to be monitored and studied for the planning of appropriate management.

At the roadsides the cover of exotic annual/biannual herbs and grasses was greater than in the reference areas, in line with results found in central Argentina [61]. Research carried out in the USA reported that the cover of exotic herbs decreased as the distance from the roadside towards the interior of the reference area increased [78]. The majority of the species that make up this group are ruderal, adapted to conditions of low stress and high disturbance levels [79], such as from the movement of soil due to highway maintenance, which is very frequent in Patagonia.

The native shrubs *Acaena splendens*, *Senecio bracteolatus* and *Azorella prolifera* had the highest levels of cover in the reference areas. This functional group was also abundant at the roadsides, being *A*. *splendens* the dominant species. This species is a ruderal shrub [*sensu* 79] that frequently colonizes sandy and degraded soils [80], and could be a key species for restoration, since it has been reported to act as a nurse species that facilitates recruitment of native seedlings of high forage value, as well as other species in arid ecosystems [81].

In the roadside seed bank, a higher density of seeds of exotic annual/biannual herbs and grasses was found than in the reference area. This functional group was dominated by *Verbascum thapsus*. The high seed density of annual herbs may be related to species traits such as fast reproduction and high production levels of long-lived, small seeds with dormancy, which tend to become buried in the soil and form persistent banks [82, 83]. *Verbascum thapsus* is categorized as invasive in other parts of the world [54, 55]. It was present in the vegetation and the seed bank of the roadsides, however in the reference areas its seeds were abundant only in the seed bank being absent in the vegetation, as this species depends on intense disturbance to spread into less degraded sites. *Verbascum thapsus* is an opportunist species that does not compete well within the natural ecosystem [84]; nevertheless, this species presents high production of small seeds that form a persistent, long-lasting bank [84].

The functional groups that contributed most seeds to the roadside seed bank were the native and exotic annual/biannual herbs and grasses and exotic perennial herbs. These functional groups were represented by diverse species that form persistent seed banks [43]. The persistent seed banks of invasive exotic species are a long-term legacy [85]. In the reference areas exotic perennial herbs and grasses and native annual herbs also dominated, represented by *R*. *acetosella* and *Heliotropium paronychioides*, respectively. The seed banks of the steppe tend to contain a high number of exotic perennial herbs [43]. Exotic shrubs were not recorded in the seed bank of roadsides and reference areas. *Rosa rubiginosa* was the only exotic shrub found in aboveground vegetation and with very low cover, therefore the probability of finding seeds in the soil is also low. The predominance of *H*. *paronychioides* in the seed banks of the roadsides and reference areas is related to the ovoid shape and small size of its seeds, which enable it to form seed banks [43]. This species was found in the seed bank but not in the vegetation of the reference areas, suggesting that its germination was favoured by the resource release produced by disturbances such as those that occur at the roadsides; it was also found in abundance after fires [86].

The index of similarity between the vegetation and the seed banks of the roadsides and reference areas analyzed in this work was higher at the roadsides than in the reference areas. When the roadsides and reference areas were compared, similarity was greater in the vegetation than in the seed banks. The high similarity between the roadside vegetation and seed bank may be due to the abundance of annual exotic and native species. Annual species have traits (short life cycles, rapid growth and long-distance dispersal) [87], which allow them to persist in very disturbed environments [88] such as roadsides. These species produce numerous small, long-lived seeds that are easily incorporated into the soil [89] contributing to the similarity between aboveground vegetation and the soil seed banks. The low similarity found between the vegetation and seed bank in the reference area follows a pattern which is common in other perennial grasslands [43, 90]. The dominant shrubs and perennial grasses in the reference area vegetation have predominantly vegetative reproduction, and usually have large seeds [91] with dispersal structures that make it difficult for them to become buried in the soil, making them easy predation targets [92]. Also, some ruderal species (e.g., *H*. *paronychioides* and *V*. *thapsus*) were found only in the seed bank, contributing to the high dissimilarity. The presence of perennial herbs and grasses only in the vegetation of the roadsides and reference areas contributed to greater similarity in the vegetation than in the seed banks.

In general, dispersal studies have reported that anemochorous species are common at roadsides [15, 23]. However, in this study the exotic and native species at the roadsides presented similar percentages of anemochorous and non-anemochorous (autochorous and zoochorous) species. This may be because various species that present autochorous dispersal (e.g., *R*. *acetosella*, *V*. *thapsus*, *H*. *paronychioides*) have small, light seeds that can also be wind dispersed and contribute mostly to the seed bank. Many exotic roadside species with long-distance dispersion mechanisms are annual herbs and grasses [23, 93]. Finally, highways may constitute corridors that channel air currents, favoring the distribution of anemochorous species along their length [69], and also non-anemochorous species [19, 20].

In conclusion, this work has demonstrated that the roadsides of the northwest Patagonian steppe constitute a reservoir of exotic species. Since the spread of exotic species is a major problem throughout the world, the importance of studying roadside vegetation and seed banks must be highlighted, in order to carry out the early identification and control of alien species, and generate ecosystem management programs.

## Supporting information

S1 File Vegetation and seed bank dataset. (XLSX)
